# DF-1-Derived exosomes mediate transmission of reticuloendotheliosis virus and resist REV-specific antibodies

**DOI:** 10.1186/s12985-024-02445-4

**Published:** 2024-08-06

**Authors:** Zhen Wang, Huizhen Cui, Yawen Zhang, Wanli Sun, Wenjie Yang, Peng Zhao

**Affiliations:** 1 College of Veterinary Medicine, Shandong Agricultural University, Tai’an, 271018 China; 2 Shandong Provincial Key Laboratory of Animal Biotechnology and Disease Control and Prevention, Tai’an, 271018 China

**Keywords:** Reticuloendotheliosis virus, Exosome, Pathogenicity, Antibody neutralization, Immune escape

## Abstract

**Background:**

*Reticuloendotheliosis* virus (REV), a member of the family Retroviridae, is a hot area of research, and a previous study showed that exosomes purified from REV-positive semen were not blocked by REV-specific neutralizing antibodies and established productive infections.

**Methods:**

To further verify the infectivity of exosomes from REV-infected cells, we isolated and purified exosomes from REV-infected DF-1 cells and identified them using Western blot and a transmission electron microscope. We then inoculated 7-day-old embryonated eggs, 1-day-old chicks and 23-week-old hens with and without antibody treatment. REV was administered simultaneously as a control.

**Results:**

In the absence of antibodies, the results indicated that REV-exosomes and REV could infect chicks, resulting in viremia and viral shedding, compared with the infection caused by REV, REV-exosomes reduced the hatching rate and increased mortality after hatching, causing severe growth inhibition and immune organ damage in 1-day-old chicks; both REV and REV-exosomes also could infect hens, however, lead to transient infection. In the presence of antibodies, REV-exosomes were not blocked by REV-specific neutralizing antibodies and infected 7-day-old embryonated eggs. However, REV could not infect 1-day-old chicks and 23-week-old hens.

**Conclusion:**

In this study, we compared the infectious ability of REV-exosomes and REV, REV-exosomes could escape from REV-specific neutralizing antibodies in embryonated eggs, providing new insights into the immune escape mechanism of REV.

**Supplementary Information:**

The online version contains supplementary material available at 10.1186/s12985-024-02445-4.

## Background

Reticuloendotheliosis is a common neoplastic disease caused by infection with the reticuloendotheliosis virus (REV), which is the third type of tumor virus after the Marek’s disease virus (MDV) and avian leukosis virus (ALV) [1]. During poultry production, co-infection with REV and other immunosuppressive viruses become more prevalent, and REV, as an exogenous virus, can contaminate live avian vaccines, which poses a significant threat to the poultry industry [2, 3].

The REV transmission route includes horizontal and vertical transmission, and REV has been detected in cock semen, which could infect offspring after artificial insemination with REV-positive semen [4]. In a previous study, we found that REV-positive semen-derived exosomes contained REV whole genome RNAs, established productive infections, and ignored antibody neutralization [5]. In recent years, exosomes have received increasing attention as an important pathway for mediating immune escape. Wang et al. verified that exosome-mediated porcine reproductive and respiratory syndrome virus (PRRSV) transmission is not completely blocked by specific neutralizing antibodies against PRRSV [6].

To further investigate the infectivity of exosomes from REV-infected cells and the ability of REV-escaped neutralizing antibodies, in this study, REV-exosomes and free REV-inoculated 7-day-old embryonated eggs, 1-day-old chicks, and 23-week-old hens with and without antibodies, compared pathogenicity and the ability of escaped antibodies, thus providing novel data on the mechanism of exosome-mediated REV-escaped immunity.

## Methods

### Cell culture and viruses

DF-1 cells were cultured in Dulbecco’s Modified Eagle Medium (DMEM; Gibco, Carlsbad, CA) with 10% heat-inactivated fetal calf serum (FBS; Gibco, Carlsbad, CA) and 1% penicillin/streptomycin in a humidified incubator at 37°C with 5% CO_2_. REV strain IBD-C1605 (GenBank accession number: KX278301) was isolated from a contaminated IBD vaccine [7]. In this study, DF-1 cells were passed and cultured overnight to 80% confluence, were infected with REV at a multiplicity of infection (MOI) of 1.0, after 3 passages of cells that were inoculated with REV, the culture supernatants were harvested and stored at -80°C, and the viral titer was measured by 50% median tissue culture infective dose (TCID_50_).

### Exosome isolation and purification

REV-exosomes or mock-infected DF-1 cell supernatants were collected and centrifuged for 5 min at 4°C to discard the cells and larger debris. The supernatant was transferred to a new tube and centrifuged at 2,000 × g for 20 min to remove cell debris. Then the supernatants were centrifuged at 10,000 × g for 30 min and filtered through a 0.22 -µm filter (Merck Millipore, USA). The filtrates were centrifuged at 10,0000 × g for 90 min at 4°C, the products were collected and suspended in 50–500 µL of particle-free phosphate-buffered saline (PBS). Exosomes were purified according to a previously published method [6].

### Transmission electron microscopy (TEM) and detection of REV whole genome

The morphology of exosomes isolated from REV-infected DF-1 cell culture supernatant was evaluated using TEM (Hitachi H-7000FA, Japan). A drop (10 µL) of exosomes was placed on a carbon-coated copper grid (200 mesh), the exosomes had an adsorption period of 1 min at room temperature, and the excess liquid was absorbed by a clean filter paper. Then, the exosome-containing grids were incubated with 1% (w/v) uranyl acetate at room temperature for 1 min, and filter paper was utilized to absorb excess uranyl acetate. The copper mesh was placed under a transmission electron microscope for evacuation.

The total DNA was isolated from the purified exosomes using a cell genome DNA extraction kit (Omega Bio-Tek, USA), according to the manufacture’s protocol. PCR was performed with TakaRa Ex taq enzyme reagent kits (TakaRa Bio, Japan), following the manufacturer’s instructions. The amplification products were detected by 1.0% agarose gel electrophoresis. Three pairs of primers used in this study according to a previously study [7].

### Western blot of exosomes and liquid chromatography-tandem mass spectrometry

Western blot was performed using the following established protocol, and total proteins were extracted from mock- or REV-exosomes using a modified RIPA buffer. The protein concentration was measured using a bicinchoninic acid assay (BCA) protein assay kit (NCM Biotech, China). Exosome lysates were subsequently loaded onto SDS-PAGE (4% stacking gel, 10% running gel), after electrophoresis, the resolved proteins were transferred onto 0.2-µm polyvinylidene difluoride (PVDF) membranes (Millipore, USA). The membrane was blocked with 5% BSA for 2 h at room temperature, then the membrane was incubated with the primary antibodies which were directed against CD63 and HSP70 (Abcam, UK) at 4°C overnight. The membranes were washed and incubated with horseradish peroxidase (HRP)-conjugated secondary antibodies, including goat anti-rabbit IgG (Beyotime Biotechnology, China). Antigen-antibody binding was visualized using enhanced chemiluminescence (ECL, Bio-Rad).

For MS analysis, Radio Immunoprecipitation Assay (RIPA) lysis and extraction buffer were added to the purified exosomes, then centrifuged the sample at 12,000 rcf for 10 min, the supernatant was collected. Then proteins were digested with FASP procedure, after digestion, the peptide was desalted using a self-priming desalting column, and the solvent was evaporated in a vacuum centrifuge at 45°C. Experiments were performed on a Q Exactive™ Hybrid Quadrupole-Orbitrap™ Mass Spectrometer (Thermo, USA) coupled to an Easy-nLC 1200 liquid chromatograph (Thermo, USA).

### Establishment and selection of REV antibody positive in embryonated eggs and chickens

Formaldehyde-inactivated REV was emulsified with an oil adjunct to produce a REV oil emulsion-inactivated vaccine. The immunization was carried out thrice 8 weeks before the first laying of SPF hens; blood samples were collected weekly after the third immunization, REV-specific antibodies were detected by Enzyme-linked immunosorbent assay (IDEXX) kit, hens with positive REV antibodies were picked, their embryonated eggs were collected, the extracted egg yolks were detected using an ELISA kit, and antibody-positive embryonated eggs were stored. A portion of embryonated eggs with REV antibody-positive hatched blood samples from 1-day-old chicks was collected to detect maternal antibodies, and chicks with positive REV antibodies were used in the later experiments.

### Infectivity of REV and REV-exosome in embryonated eggs

To further compare the infectivity of different infections, REV exosomes or free REV at the same TCID_50_ (500) were used to infect 7-day-old embryonated eggs with and without antibodies. Blood and cloacal swabs were collected at 1, 7, 14, and 21 day post infection after hatching, and REV was detected by quantitative real-time PCR. The total RNAs of samples were extracted with the E.Z.N.A total RNA kit I (Omega Bio-tek), the isolated RNA was reverse transcribed into cDNA using M-MLV RT Kit (TakaRa) per the manufacturer’s protocol, quantitative real-time PCR was performed with SYBR Green Premix Pro Taq HS qPCR Kit (Accurate Biology, China), the primers used to quantify the REV were as follows: 5′-CCCCATTCATGTCCAGCTAT-3′ and 5′-AGGGAGGAGAGGAGTGTTCC-3.’

### Infectivity of REV and REV-exosome in chickens

REV-exosome or free REV at the same TCID_50_ (1000) were used to infect 1-day-old chicks with and without antibodies, blood, cloacal swabs, and immune organs were collected at 7, 14 and 21 dpi, the spleen, bursa, and thymus were weighted and stored, the mRNA levels of IFN-α, IFN-β, IFN-γ and Mx genes in liver and spleen were detected and analyzed. Primers used for real-time polymerase chain reaction (PCR) shown in Table 1. The SPSS version 25 was used for statistical analysis. The relative quantification of gene expression was normalized by normalization to the level of β-actin, and the expression levels of genes in the REV-infected group were arbitrarily set to 1. Utilized the 2^−ΔΔCt^ method to analyze the relative RNA expression levels. Data were analyzed and plotted using the GraphPad Prism 6 software.

**Table 1:** Primers used for real-time PCR.

	Primer sequence	GenBank accession no.	Product size (bp)
REV-1	F: 5′-AATGTGGGAGGGAGCTCTGGGGGGA-3′	KX278301	1798
	R: 5′-CCTGATCATTCCTGACCTCCCGCC-3′		
REV-2	F: 5′-ACCAGACTCTACTGTAATGGCGAGCC-3′	KX278301	3401
	R: 5′-AATACTGAGGGGTTTCGGTATCTGG-3′		
REV-3	F: 5′-TCTGGCTACCTAAACGGGTAGCTGT-3′	KX278301	3660
	R: 5′-CCCCCAAATGTTGTACCGAAATACTA-3′		
IFN-α	F: 5’-CGCAACCTTCACCTCACCATCAAA-3’	NM-205427.1	93
	R: 5’-TGTGAGGTTGTGGATGTGCAGGAA-3’		
IFN-β	F: 5’-GCCCACACACTCCAAAACACTG-3’	KF-741874.1	151
	R: 5’-TTGATGCTGAGGTGAGCGTTG-3’		
IFN-γ	F: 5’-AAGTCAAAGCCGCACATCAAAC-3’	NM-205149.1	132
	R: 5’-CTGGATTCTCAAGTCGTTCATCG-3’		
Mx	F: 5’-CAGCTCCAGAATGCATCAGA-3’	XM-046906529.1	156
	R: 5’-GGCAATTCCAGGAAGATCAA-3’		
β-actin	F: 5’-GAGAAATTGTGCGTGACATCA-3’	NM-205518.2	152
	R: 5’-CCTGAACCTCTCATTGCCA-3’		

REV-exosomes or free REV at the same TCID_50_ (10,000) were used to infect 23-week-old hens with and without antibodies, blood and cloacal swabs were collected at 7, 14, 21, 28, and 35 dpi, and REV was detected by quantitative real-time PCR.

## Results

### Isolation, identification, and component analysis of REV-infected DF-1 cells

**Fig. 1:**
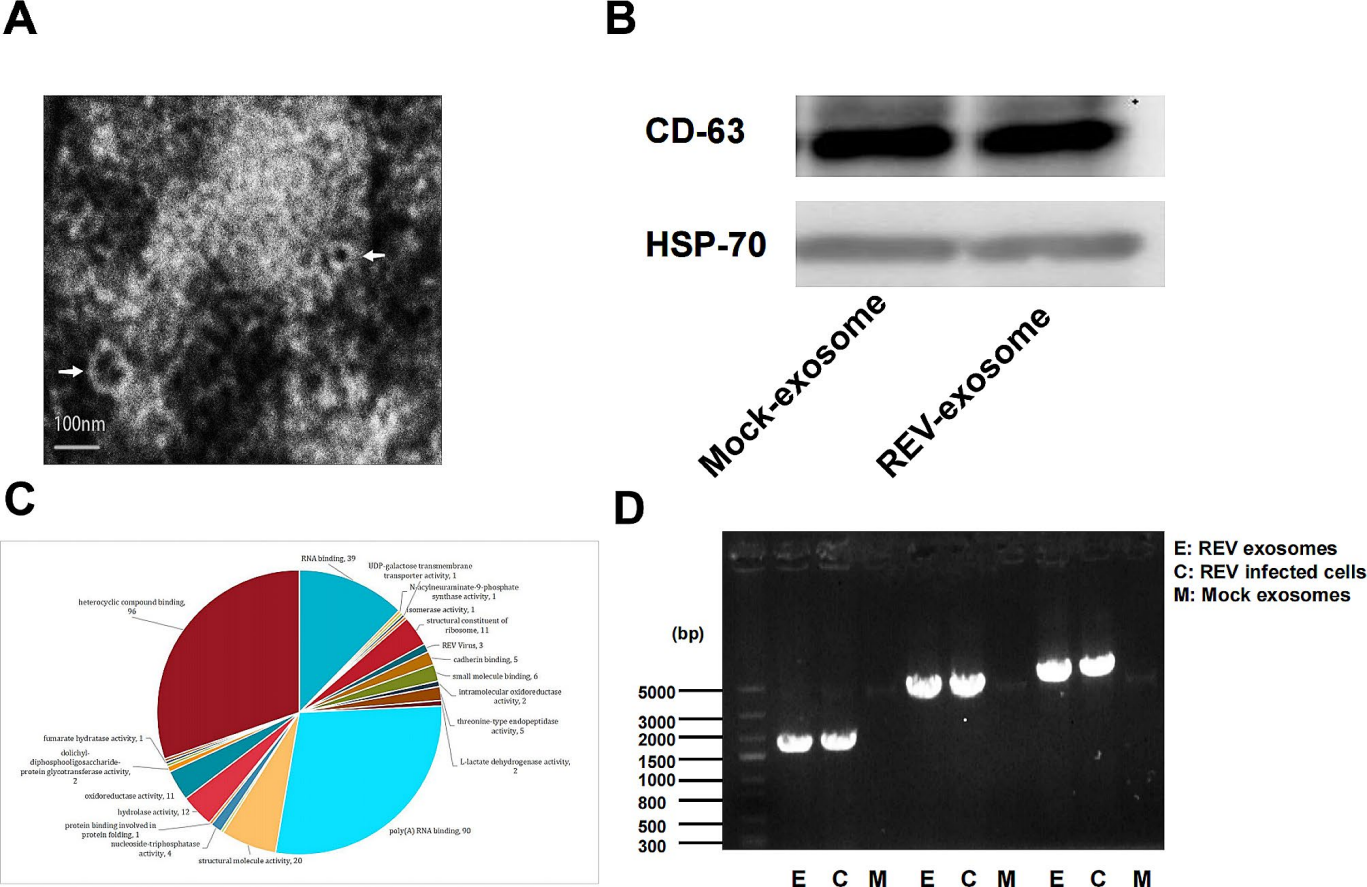
Isolation and characterization of exosomes from REV-infected DF-1 cells. (**A**) Transmission electron microscopy observation of negatively stained the exosome, scale bar = 100 nm. (**B**) Purified exosomes derived from mock- or REV-infected DF-1 cells were analyzed on Western blot with antibodies against CD63 and HSP70. (**C**) LC-MS/MS analysis of the purified exosomes from REV-infected DF-1 cells, to detect the presence of REV proteins in exosomes. (**D**) Three pairs of primers designed against REV genome sequence in purified exosomes isolated from REV-infected DF-1 cells, the targeted fragments were amplified with sizes of 1798, 3401 and 3632 bp

The cell supernatant was collected 24 h after REV-infected DF-1 cells, and exosomes were isolated through differential centrifugation, TEM demonstrated that exosomes displayed a cup-shaped appearance ranging from about 100 nm in size (Fig. 1A), which conformed to the size and morphology of exosomes, Western blot was used to analyze the exosomal markers, two representative exosome markers, CD63 and HSP70 were detected (Fig. 1B). These results indicated that the exosomes were successfully isolated. Furthermore, liquid chromatography-tandem mass spectrom spectrometry (LC-MS/MS) found the three REV-related proteins, namely, pol-gag polyprotein, env and REV-polymerase (Fig. 1C). The whole-genome of REV was detected in the purified exosomes from REV-infected DF-1 cells (Fig. 1D).

### Lethality of REV exosomes and REV on embryonated eggs

Fifty embryonated eggs were divided into two groups: 10 embryonated eggs hatched in the REV-exosome group, with a hatching rate of 40%, and 17 embryonated eggs hatched in the REV group, with a hatching rate of 68% (Table 2). REV-exosomes significantly reduced the hating rate (*P* < 0.05). Fifty embryonated eggs with antibodies were divided into two groups: 9 embryonated eggs hatched in the REV-exosome group, with a heating rate of 36%, and 21 embryonated eggs hatched in the REV group, with a hatching rate of 84% (Table 2). REV was detected in all dead embryonated eggs. The results showed that REV could be blocked by REV-specific neutralizing antibodies, REV-exosomes could infect embryonated eggs and resist neutralizing antibodies.

**Table 2:** Hatching rate of 7-day-old embryonated eggs infected with REV-exosome or REV

Groups	embryonated eggs without antibodies	embryonated eggs with antibodies
REV-exosome group	40.0% (10/25)	36.0% (9/25)
REV group	68.0% (17/25)	84.0% (21/25)

### Inoculation of REV exosomes into 7-day-old embryonated eggs resulted in viremia and viral shedding

**Fig. 2:**
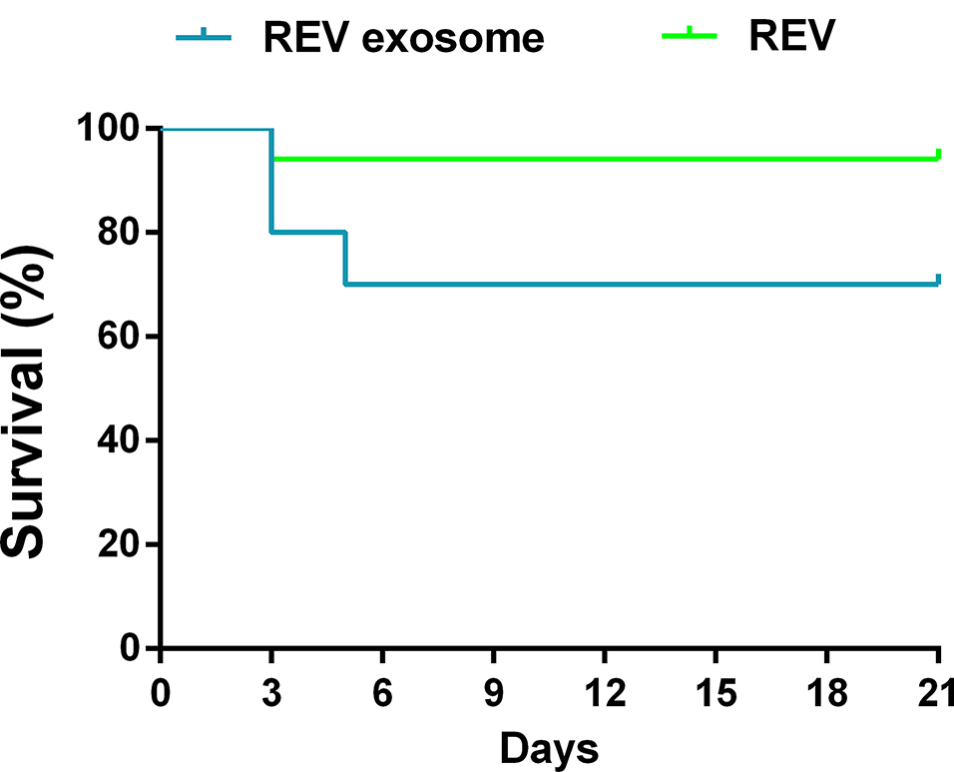
Seven-day-old embryonated eggs were infected with REV-exosomes or REV after hatching, and the chick survival rate was determined

7-day-old embryonated eggs without antibodies were infected with REV or REV-exosome, and after hatching, the REV group died a chick at 3 d, REV-exosome group died 2 chickens at 3 d, died a chick at 5 d (Fig. 2), all dead chicks detected REV through qPCR. REV and REV-exosomes did not cause death after embryonated eggs hatched with antibodies.

REV-exosomes or free REV were used to infect 7-day-old embryonated eggs without antibodies and viremia was detected at 1, 7, 14, and 21 dpi; the positive rate of the REV-exosome group was higher than that of the REV group, and the REV-exosome group maintained a high positive rate for a long time (Table 3), suggesting that the REV-exosome group had stronger infectivity than in the REV group, and viral shedding was detected in the cloacal swab of the REV and REV-exosome groups (Table 3). We infected 7-day-old embryonated eggs with antibodies with REV or REV-exosomes, and viremia was detected at 1, 7, 14, and 21 dpi. In the presence of antibodies, REV-exosomes also infected embryonated eggs and maintained a high positive rate (Table 4). Simultaneously, cloacal swabs were also tested (Table 4). These results suggest that exosome-mediated REV infection could not be blocked by REV-specific neutralizing antibodies. However, REV did not infect embryonated eggs with antibodies, and viral shedding was not found in the REV group.

**Table 3:** Detection of viremia and cloacal swabs from embryonated eggs without antibodies

Time (days)	Positive rate of REV in blood		Positive rate of REV in cloacal swabs	
	REV-exosome	REV	REV-exosome	REV
1 d	80.0% (8/10)	58.8% (10/17)	80.0 (8/10)	23.5% (4/17)
7 d	85.7% (6/7)	56.3% (9/16)	85.7% (6/7)	43.8% (7/16)
14 d	100% (7/7)	56.3% (9/16)	57.1% (4/7)	62.5% (10/16)
21 d	100% (7/7)	37.5% (6/16)	28.6% (2/7)	100% (16/16)

**Table 4:** Detection of viremia and cloacal swabs from embryonated eggs with antibodies

Time (days)	Positive rate of REV in blood		Positive rate of REV in cloacal swabs		
	REV-exosome	REV		REV-exosome	REV
1 d	33.3%(3/9)	0%(0/21)		33.3%(3/9)	0%(0/21)
7 d	66.7%(6/9)	0%(0/21)		66.7%(6/9)	0%(0/21)
14 d	66.7%(6/9)	0%(0/21)		66.7%(6/9)	19.0%(4/21)
21d	66.7%(6/9)	0%(0/21)		33.3%(3/9)	0%(0/21)

### Infectivity and pathogenicity of REV-exosome and REV in 1-day-old chicks

**Fig. 3:**
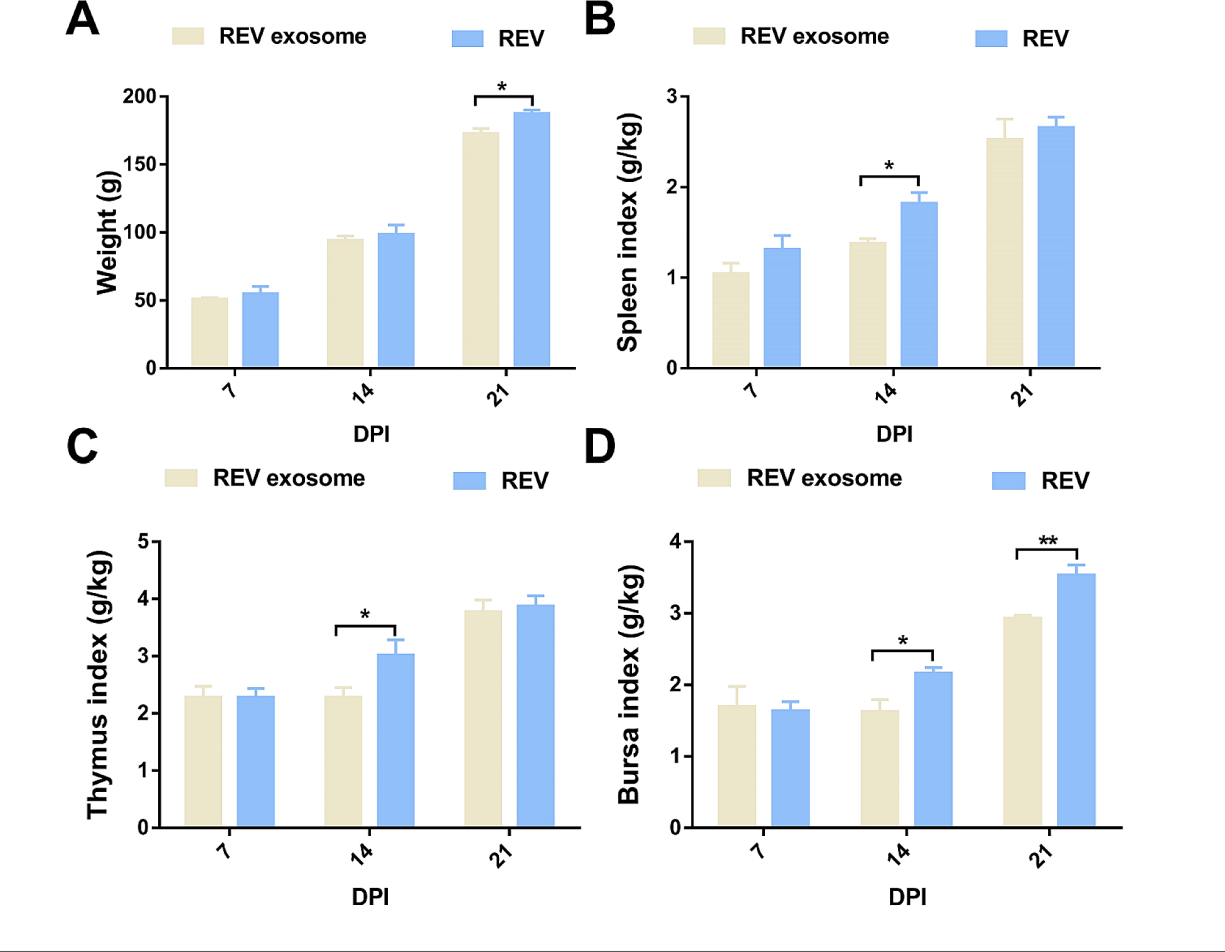
One-day-old chicks were infected with REV-exosomes or REV, and the body weight and immune organ index were recorded. The body weights of the infected chicks were recorded at 7, 14, and 21 dpi. The whole spleen, thymus, and bursa from the infected chicks were collected at 7, 14, and 21 dpi, weighed, and recorded. The body weights of the infected chicks were recorded at 7, 14, and 21 dpi. (**A**) Changes in body weight from 1 to 21 dpi. (**B**–**D**) Changes in immune organ index from 1 to 21 dpi. * *p* < 0.05, ** *p* < 0.01, *** *p* < 0.001

REV- or REV-exosome-infected 1-day-old chicks without antibodies and detection of viremia at 7, 14, and 21 dpi showed no differences in the early stages of infection. However, with an increasing number of days, the REV-exosome group was all infected, but the positive rate of the REV group decreased (Table 5). REV-exosomes and REV resulted in viral shedding as detected by cloacal swabs (Table 5). REV- and REV-exosome-infected 1-day-old chicks with antibodies, viremia, or viral shedding were not detected. Compared to the REV group, the REV-exosome group resulted in more severe growth inhibition and immune organ damage in chicks. The body weight of REV group (Fig. 3A) was significantly higher than that of REV-exosome group at 21 dpi (*P* < 0.05), the spleen index (Fig. 3B) of REV-exosome group was significantly lower than group REV (*P* < 0.05), the thymus index (Fig. 3C) of REV-exosome group was significantly lower than REV group at 14 dpi (*P* < 0.05), and the bursa index (Fig. 3D) of REV-exosome group was significantly lower than REV group at 14 and 21 dpi (*P* < 0.05, *P* < 0.01). These results indicated that REV-exosome induced more severe atrophy of the thymus, spleen, and bursa. In 1-day-old chicks with antibodies, no differences were observed between the REV-exosome and REV groups.

**Table 5:** Detection of viremia and cloacal swabs from 1-day-old chicks without antibodies

Time (days)	Positive rate of REV in blood		Positive rate of REV in cloacal swabs	
	REV-exosome	REV	REV-exosome	REV
7 d	80.0%(12/15)	80.0%(12/15)	80.0%(12/15)	80.0%(12/15)
14 d	100%(12/12)	100%(12/12)	83.3%(10/12)	41.7%(5/12)
21 d	100%(9/9)	55.6%(5/9)	55.6%(5/9)	77.8%(7/9)

### Detection of immune-related genes after REV-exosome or REV-infected 1-day-old chicks

**Fig. 4:**
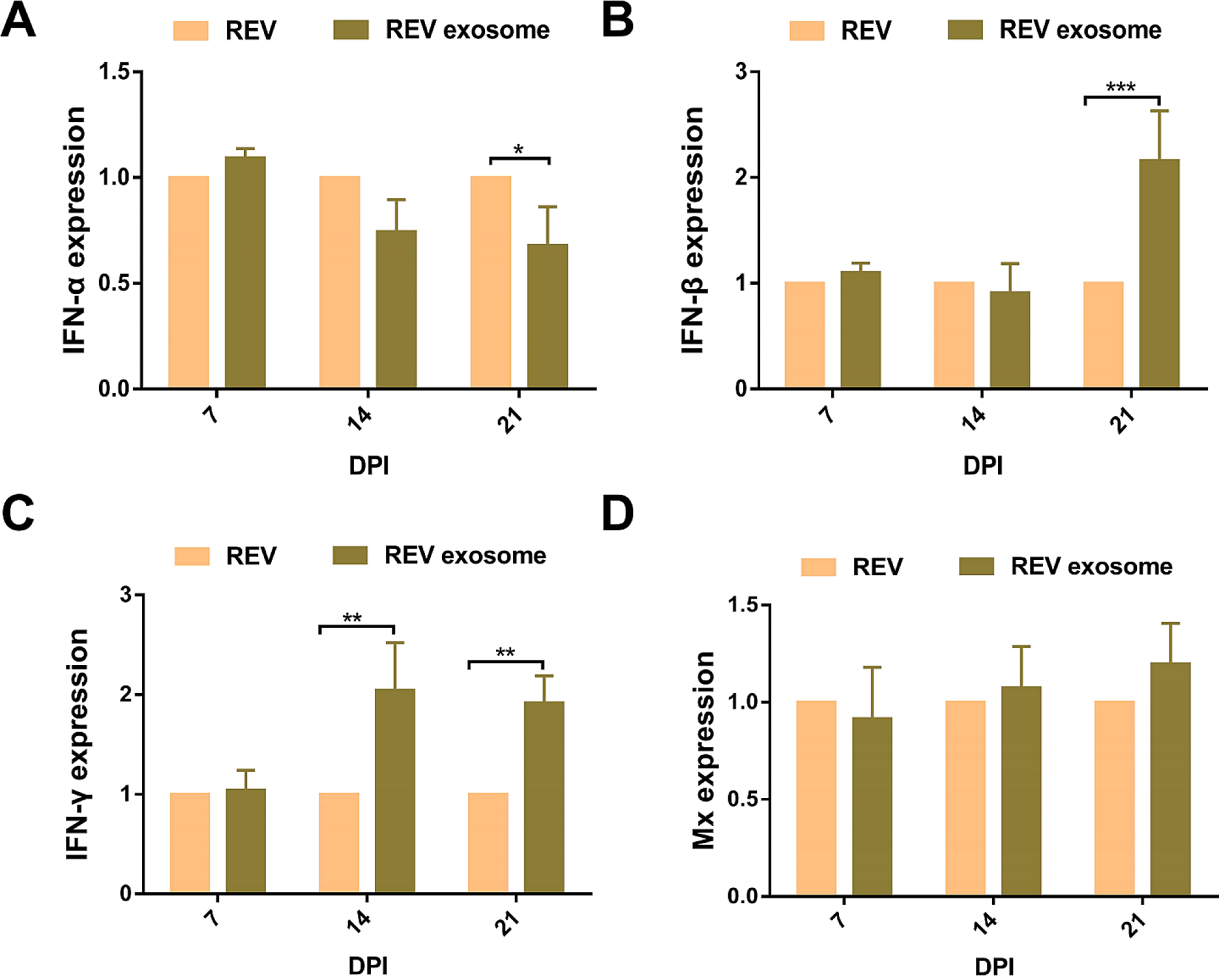
mRNA levels of immune-related genes in the spleens of infected chicks. 1-day-old chicks were infected with REV-exosome or REV, and spleen samples were collected at 7, 14, and 21 dpi. The immune-related genes in the spleens were detected by real-time quantitative reverse transcription polymerase chain reaction analysis, and relative expression levels were normalized to the β-actin gene and calculated using the 2^-ΔΔCt^ method. (**A**–**D**) IFN-α, IFN-β, IFN-γ and Mx expression levels. * *p* < 0.05, ** *p* < 0.01, *** *p* < 0.001

**Fig. 5:**
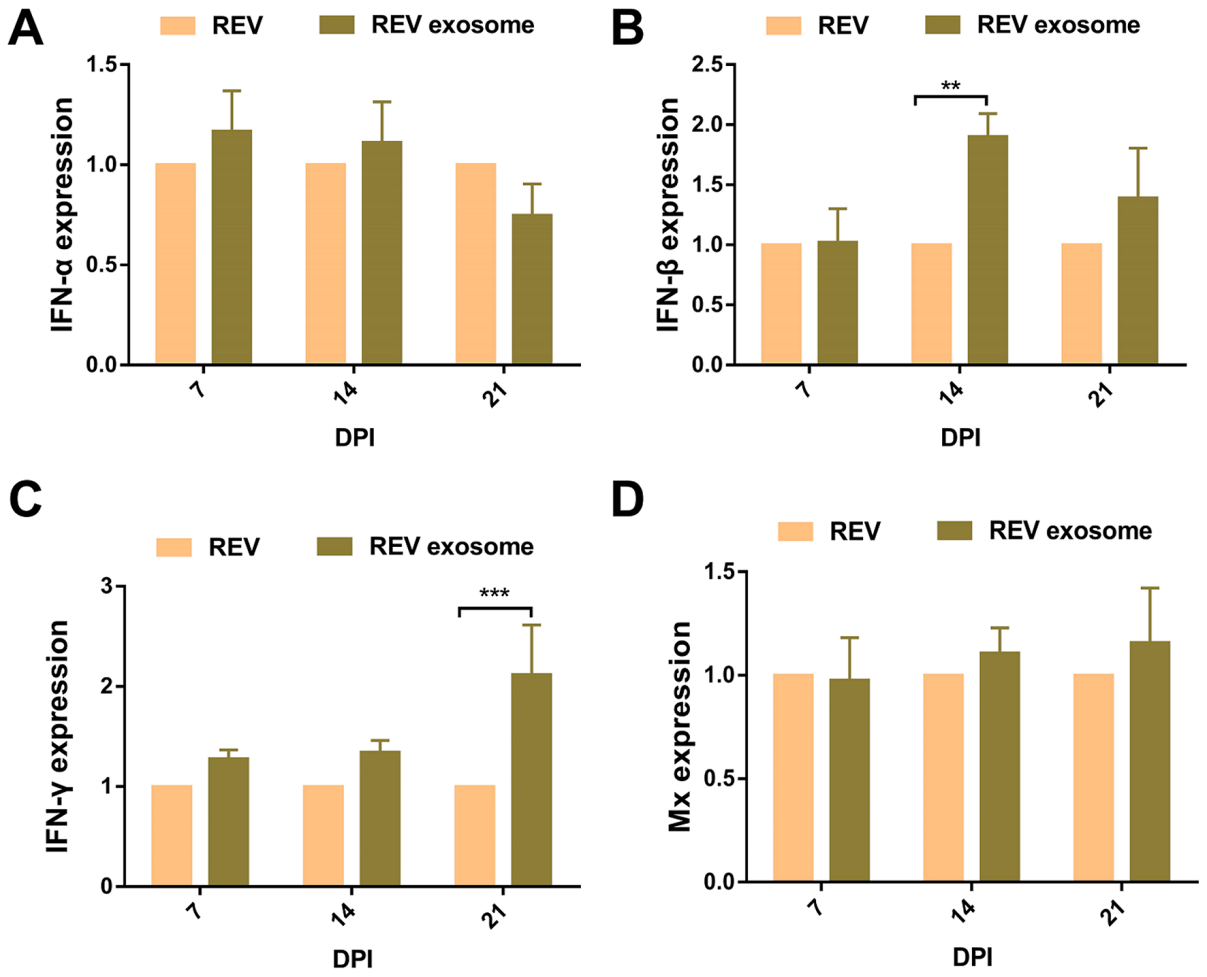
mRNA levels of immune-related genes in the livers of infected chicks. 1-day-old chicks were infected with REV-exosome or REV, and liver samples were collected at 7, 14, and 21 dpi. The immune-related genes in the livers were detected by real-time quantitative reverse transcription polymerase chain reaction analysis, and relative expression levels were normalized to the β-actin gene and calculated using the 2^− ΔΔ Ct^ method. (**A**–**D**) IFN-α, IFN-β, IFN-γ and Mx expression levels. * *p* < 0.05, ** *p* < 0.01, *** *p* < 0.001

REV or REV-exosome infected 1-day-old chicks without antibodies and their liver and spleen samples collected at 7, 14, and 21 dpi, and the IFN-α, IFN-β, IFN-γ, and Mx mRNA levels were detected using qRT-PCR. In the spleen, the IFN-α mRNA level (Fig. 4A) in the REV-exosome group was significantly lower than this in the REV group at 21 dpi (*P* < 0.05), the IFN-β mRNA level (Fig. 4B) in the REV-exosome group was significantly higher at 21 dpi (*P* < 0.001), and the IFN-γ mRNA levels (Fig. 4C) in REV-exosome group was significantly higher at 14 and 21 dpi (*P* < 0.01). However, there was no difference in the Mx mRNA levels (Fig. 4D) between the REV-exosome group and the REV group. At the same time, we plot the expression levels of both groups over time in the spleen, each group used the cytokine mRNA levels on day 7 as a control, the IFN-α mRNA levels of REV-exosome group (Fig S4A) was significantly decreased at 21 dpi (*P* < 0.05). The IFN-α and IFN-β mRNA levels of REV group (Fig S4E and F) were also significantly decreases at 21 dpi (*P* < 0.05, *P* < 0.01). In the liver, the IFN-α (Fig. 5A) and Mx (Fig. 5D) mRNA levels were not significantly different, the IFN-β (Fig. 5B) mRNA level in the REV-exosome group was significantly higher than this of the REV group at 14 dpi (*P* < 0.01), the IFN-γ (Fig. 5C) transcription level was significantly higher at 21 dpi (*P* < 0.001). We also plot the expression levels of both groups over time in the liver, each group used the cytokine mRNA levels on day 7 as a control, the IFN-α, IFN-β and IFN-γ mRNA levels (Fig S5A, B and C) were significantly upregulated in REV-exosome group at 14 dpi (*P* < 0.05, *P* < 0.01, *P* < 0.05), IFN-α and IFN-β mRNA levels (Fig S5A and B) were significantly decreased at 21 dpi (*P* < 0.01, *P* < 0.05). In REV group, the IFN-α and IFN-γ mRNA levels (Fig S5E and G) were significantly upregulated (*P* < 0.01, *P* < 0.05) ,the IFN-α, IFN-γ and Mx mRNA levels (Fig S5E, F and H) were significantly decreased (*P* < 0.01, *P* < 0.01, *P* < 0.01). The results suggested that the REV-exosome-induced immune response was stronger than that of REV. In the early stage of infection, exosome-mediated REV infection was not recognized by the immune system, which facilitated the replication and proliferation of viruses, whereas in the later stage of infection, massive proliferation of the virus resulted in strong immune responses. Through ploting the expression levels of both groups over time, we found that two groups showed similar trends in cytokine changes, which may be related to viral proliferation.

### Susceptibility of REV-exosome and REV in 23-week-old hens

REV-exosome and REV infected 23-week-old hens without antibodies, viremia was detected at 7, 14, and 21 dpi, and both REV-exosome and REV could infect hens for a period of time (Table 6), leading to transient infection. Viral shedding was not detected in the cloacal swabs (Table 6). REV-exosome and REV-infected 23-week-old hens with antibodies, viremia, or viral shedding were not detected.

**Table 6:** Detection of viremia and cloacal swabs from 23-week-old hens without antibodies

Time (days)	Positive rate of REV in blood		Positive rate of REV in cloacal swabs	
	REV-exosome	REV	REV-exosome	REV
7 d	0%(0/15)	0%(0/15)	0%(0/15)	0%(0/15)
14 d	60%(9/15)	40%(6/15)	0%(0/15)	0%(0/15)
21 d	0%(0/15)	0%(0/15)	0%(0/15)	0%(0/15)

## Discussion

Even if the host immune system makes a response and produces neutralizing antibodies, certain viruses can hijack secretory pathways in extracellular vesicles to escape antibody recognition, Johnstone et al. named these vesicles “exosomes” [8], the exosomes play important roles in intercellular communication and signal transduction [9–11]. Retrovirus utilized formation and secretory pathways of exosomes in host cells to produce infectious virus particles, productive infection was established by receptors independent pathway [12]. Exosomes derived from hepatitis C virus (HCV)-infected cells carry viral RNA and can mediate the viral-receptor-independent transmission of HCV [13]. Exosomes isolated from human immunodeficiency virus (HIV)-infected cells make the recipient cells more susceptible to HIV infection [14]. In recent years, exosomes have received more attention as an important pathway for mediating immune escape, and our previous studies indicated that REV-positive semen-derived exosomes not only included REV whole-genome RNAs but also established productive infection and escaped neutralizing antibodies [5]. Exosomes from CEF infected with ALV-J or REV contained various viral genes and structural proteins [15]. This study aimed to determine whether exosomes from cells infected with REV establish productive infections in embryonated eggs, chicks, and hens and whether exosomes establish productive infection by evading the clearance of neutralizing antibodies.

In this study, we found that REV utilized exosomes to establish productive infections and has stronger pathogenicity. To determine exosome infectivity, we first utilized the extracted exosomes to infect 7-day-old embryonated eggs, 1-day-old chicks, and 23-week-old hens, which confirmed that exosomes from cells infected with REV resulted in the infection of embryonated eggs and chicks, which had strong infectivity. Why does REV-exosome group had strong infectivity? We think that exosomes are not easily recongnized by the immune system, in the early stages of viral infection, REV-exosome evade the body’s immune surveillance, therefore, REV can more effectively replicate and proliferate. Compared to REV, REV-exosome has stronger pathogenicity.

When REV faced the blocking effect of REV-specific neutralizing antibodies, we investigated whether exosomes from cells infected with REV established productive infection in embryonated eggs, chicks, and hens and whether they established productive infection by evading the clearance of neutralizing antibodies. As an important pathway for immune escape, exosome-mediated viral infection cannot be blocked by specific neutralizing antibodies [6]. In this study, we found that REV-exosomes could infect embryonated eggs with antibodies, but REV was blocked by specific neutralizing antibodies and therefore could not infect embryonated eggs. The exosomes are a type of extracellular vesicle, many studies have showen that viruses could utilize EVs for cell-to-cell transmission [16–22], this strategy can help many virus to evade host immune surveillance and antibody neutralization. Gao et al. proved that apoptotic bodies (ApoBDs)-mediated viral transmission is fully resistant to swine sera to ASFV [23]. Our research also proved that REV-exosomes resist to antibody neutralization.

Compared to the REV group, the IFN-β and IFN-γ mRNA levels were significantly increased in the liver and spleen of the REV-exosome group, but in the early stage of infection, there were no differences in the immune responses between the REV-exosome group and the REV group. We hypothesized that REV-exosomes evade immune system surveillance in the early stages of infection, facilitating viral replication and proliferation. This massive viral proliferation induces a strong immune response and elevated cytokine levels. We plot the expression levels of both groups over time in the spleen and liver, the two groups were increased earlier and decreased later, showing the same trend, this may be related to proliferation of the virus.

## Conclusion

This study confirmed that REV-exosomes could establish a productive infection in embryonated eggs; 1-day-old chicks and 23-week-old hens had a stronger pathogenicity than REV. Exosome-mediated REV infection was not blocked by REV-specific neutralizing antibodies; thus, 7-day-old embryonated eggs were infected with REV and chicks carried REV for a long time. This study further enrichs the data on exosomes enhancing the REV infectivity and provids novel insights into the REV immune escape mechanism.

### Electronic supplementary material

Below is the link to the electronic supplementary material.

Supplementary Material 1: **Figure supplement 1**. Transmission electron microscopy observation of negatively stained the exosome.

Supplementary Material 2: The western blot with antibody against CD63.

Supplementary Material 3: The western blot with antibody against HSP70.

Supplementary Material 4: **Figure supplement 4**. mRNA levels of immune-related in the spleen. Each group used the cytokine mRNA levels on day 7 as a control, relative expression levels were normalized to the β-actin gene and calculated using the 2^−ΔΔCt^ method. (A-D) IFN-α, IFN-β, IFN-γ and Mx expression levels of REV-exosome group. (E-H) IFN-α, IFN-β, IFN-γ and Mx expression levels of REV group. * p<0.05, ** p<0.01, *** p<0.001.

Supplementary Material 5: **Figure supplement 5**. mRNA levels of immune-related in the liver. Each group used the cytokine mRNA levels on day 7 as a control, relative expression levels were normalized to the β-actin gene and calculated using the 2^−ΔΔCt^ method. (A-D) IFN-α, IFN-β, IFN-γ and Mx expression levels of REV-exosome group. (E-H) IFN-α, IFN-β, IFN-γ and Mx expression levels of REV group. * p<0.05, ** p<0.01, *** p<0.001.

## Data Availability

No datasets were generated or analysed during the current study.

## Abbreviations

REV: Reticuloendotheliosis virus
MDV: Marek’s disease virus
ALV: Avian leukosis virus
DMEM: Dulbecco’s Modified Eagle Medium
FBS: Fetal calf serum
IBD: Infectious bursal disease
TCID50: 50% median tissue culture infective dose
TEM: Transmission electron microscope
PVDF: Polyvinylidene difluoride
LC-MS/MS: Liquid chromatography-tandem mass spectrom spectrometry
CEF: Chicken embryo fibroblasts
