# Supplementary figures and images for: DF-1-Derived exosomes mediate transmission of reticuloendotheliosis virus and resist REV-specific antibodies
Source: Virol J. 2024 Aug 6;21:177. doi: 10.1186/s12985-024-02445-4 (PMC11304787; doi:10.1186/s12985-024-02445-4)

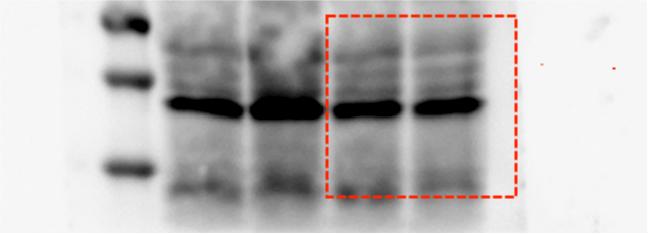

Supplement: Supplementary file 1 — Supplementary Material 1: Figure supplement 1. Transmission electron microscopy observation of negatively stained the exosome. [file 12985_2024_2445_MOESM1_ESM.tif]

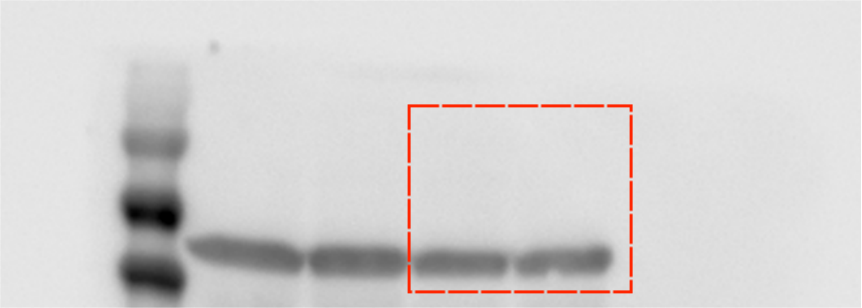

Supplement: Supplementary file 2 — Supplementary Material 2: The western blot with antibody against CD63. [file 12985_2024_2445_MOESM2_ESM.tif]

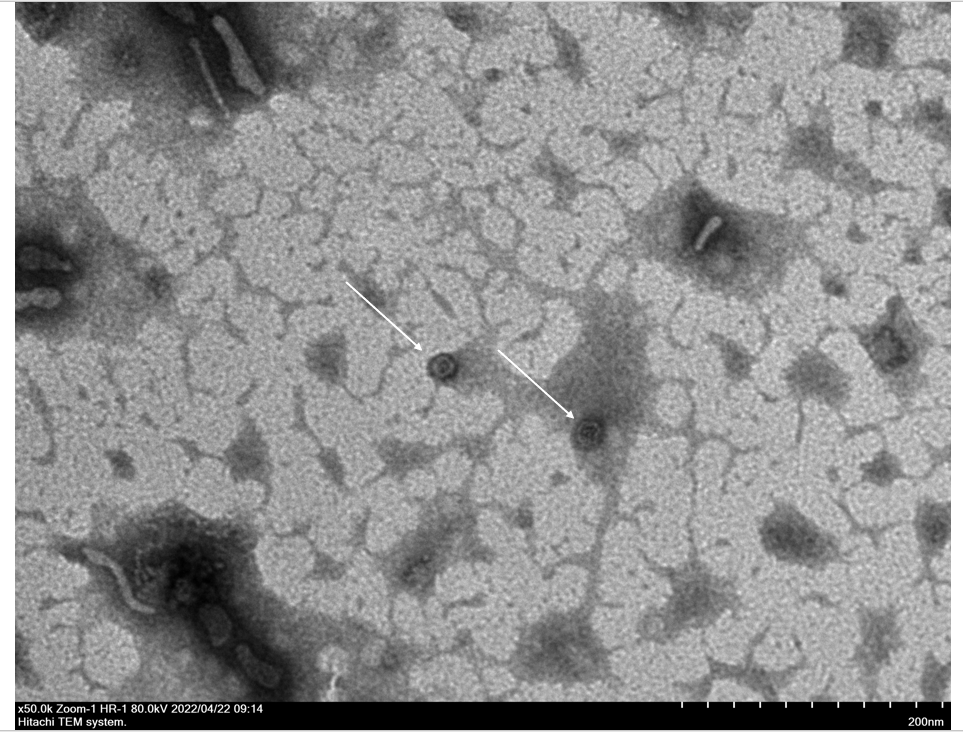

Supplement: Supplementary file 3 — Supplementary Material 3: The western blot with antibody against HSP70. [file 12985_2024_2445_MOESM3_ESM.tif]

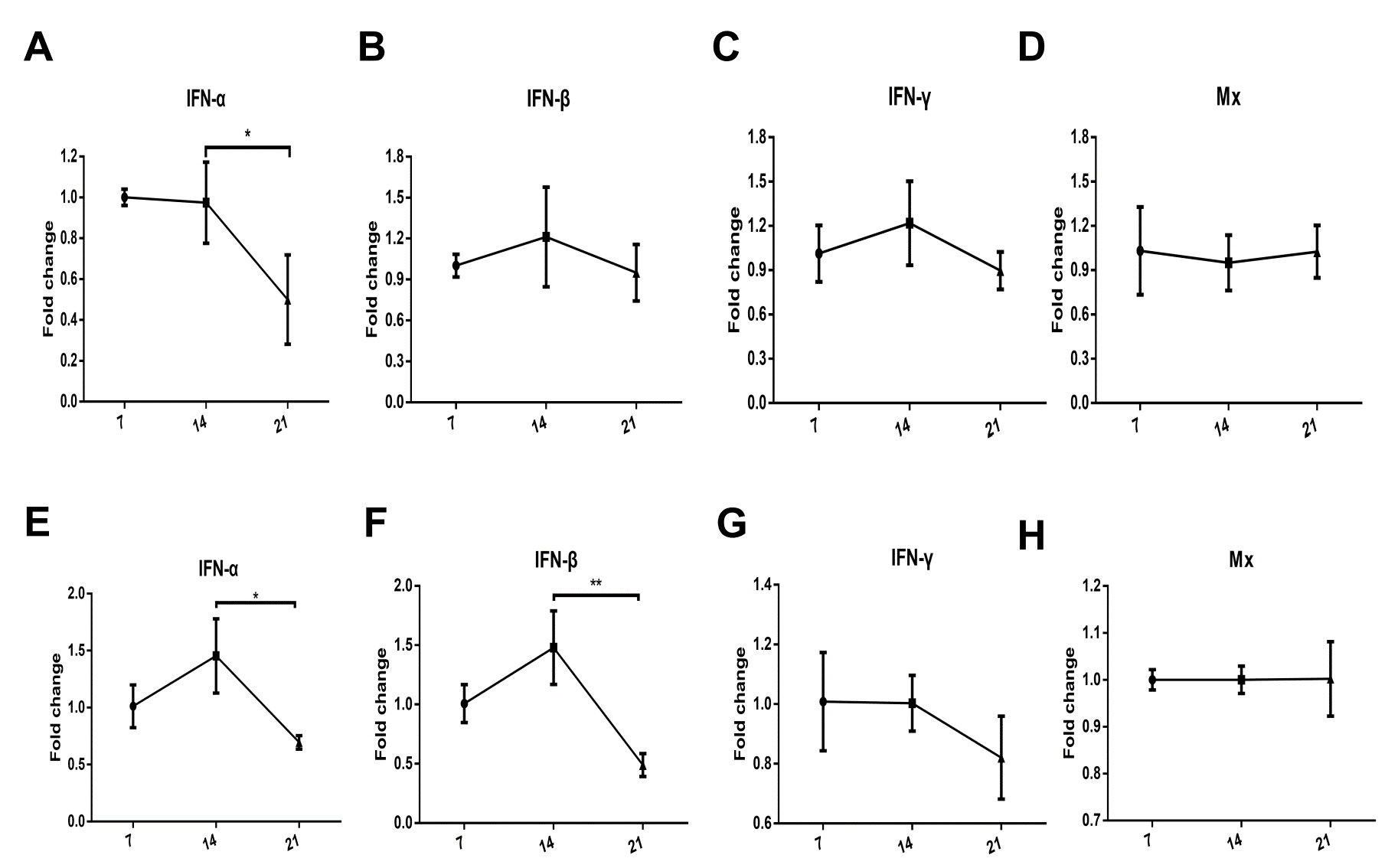

Supplement: Supplementary file 4 — Supplementary Material 4: Figure supplement 4. mRNA levels of immune-related in the spleen. Each group used the cytokine mRNA levels on day 7 as a control, relative expression levels were normalized to the β-actin gene and calculated using the 2−ΔΔCt method. (A-D) IFN-α, IFN-β, IFN-γ and Mx expression levels of REV-exosome group. (E-H) IFN-α, IFN-β, IFN-γ and Mx expression levels of REV group. * p<0.05, ** p<0.01, *** p<0.001. [file 12985_2024_2445_MOESM4_ESM.tif]

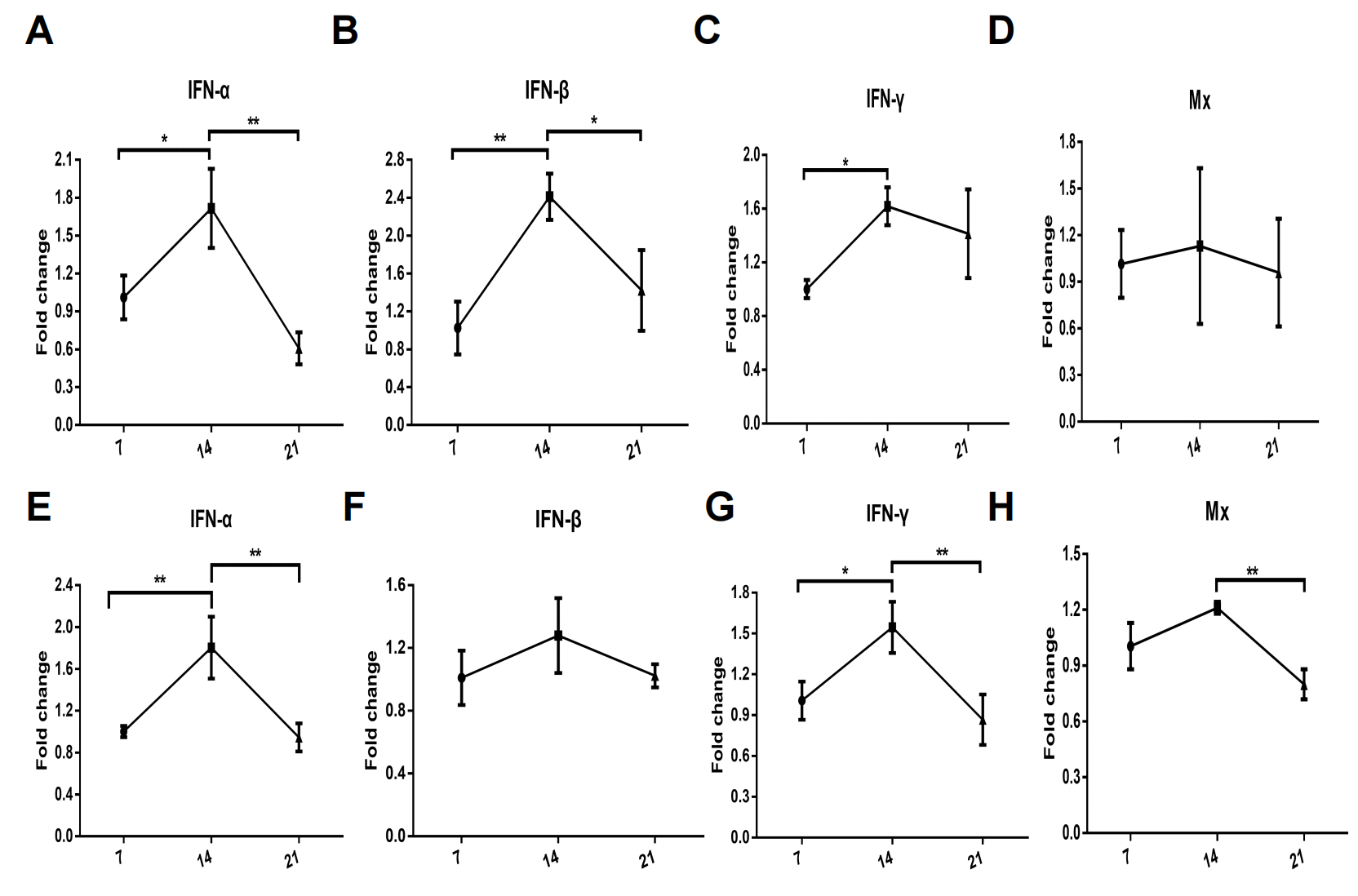

Supplement: Supplementary file 5 — Supplementary Material 5: Figure supplement 5. mRNA levels of immune-related in the liver. Each group used the cytokine mRNA levels on day 7 as a control, relative expression levels were normalized to the β-actin gene and calculated using the 2−ΔΔCt method. (A-D) IFN-α, IFN-β, IFN-γ and Mx expression levels of REV-exosome group. (E-H) IFN-α, IFN-β, IFN-γ and Mx expression levels of REV group. * p<0.05, ** p<0.01, *** p<0.001. [file 12985_2024_2445_MOESM5_ESM.tif]
